# 

**Published:** 1916-11-04

**Authors:** 


### CONTENTS.

| | PAGE |
|---|---|
| **The Selection of Hospital Staffs...** | 77 |
| **Knighthoods for Hospital Secretaries ...** | 78 |
| **Hospital and Institutional News ...** | 79 |

One of Florence Nightingale's Doctors—Memorial to an Irish Surgeon—Hospital Architecture—Military Hospital Construction—A Medical Missionary Exhibition—A Scathing Report—The Board of Control's Severe Censure—The North Wales Asylum and the Board of Control—Retirement of a Mental Hospital Superintendent—Serious Hospital Fire in Canada—A Welsh Orthopædic Hospital—Sanatoria after the War—The Rise of Institutional Salaries—Great Effort by a High Sheriff—A Good Family Record—A State Medical Service—For and Against—The Trade in Abortifacients—The Sundering Cinema—The Anomalous Position of the Military Chaplain—A Surcharge Remitted—Juvenile Employment—Promising the Impossible—Alleged Hospital Abuse in New Zealand—The Welsh National Memorial Association—The Prairie Nurse—Military Hospital Expansion at Carlisle—Death of a Hospital Treasurer—Two New Medical V.C.s—This Week's Drug Market—To our Readers.

| | |
|---|---|
| **The Hospital and the Surgeon ...** | 85 |

I.—The Selection of the Staff.

| | |
|---|---|
| **Life and Death: Some Human Lessons in War-Time ...** | 87 |

VI.—When Life is and is not a Failure.

| | PAGE |
|---|---|
| **Philosophy in Hospital Architecture ...** | 89 |
| **Military Hospital Construction ...** | 93 |
| **A Group of Plans ...** | 97 |

The New Arbroath Infirmary.
Leicester Royal Infirmary Children's Hospital.
Derbyshire Sanatorium, Walton, Chesterfield.
The Peter Bent Hospital, Boston, Mass.
Port Sunlight Cottage Hospital.

| | |
|---|---|
| **The Matrons' and Sisters' Department ...** | 103 |

Poor-Law Institutions and Nurse-Training.
Round the Hospitals.
Mrs. Fenwick Recalcitrant—Presentation to Miss Donaldson—Promotion in Q.A.I.M.N.S.—Poor-Law Matrons' Referendum—District Nursing at Liverpool—The Massage Schism.

| | |
|---|---|
| **The Heating of Hospitals ...** | 105 |

Ham Green Hospital and Sanatorium, Bristol.

| | |
|---|---|
| **The Editor's Letter-Box ...** | 107 |

Military Hospital Construction—The District Nurse.

| | |
|---|---|
| **Hospital and Institutional Results in 1915 ...** | 107 |
| **Parliament and the Profession ...** | 108 |
| **Institutional Needs ...** | xviii |
| **Matrons' and Sisters' Appointments ...** | xviii |
| **The Institutional Worker ... (Suppl.)** | 1 |

The Supply of Comforts for Wounded and Warriors.

